# A Longitudinal Study of Immune Cells in Severe COVID-19 Patients

**DOI:** 10.3389/fimmu.2020.580250

**Published:** 2020-10-23

**Authors:** Didier Payen, Maxime Cravat, Hadil Maadadi, Carole Didelot, Lydia Prosic, Claire Dupuis, Marie-Reine Losser, Marcelo De Carvalho Bittencourt

**Affiliations:** ^1^ Université Paris 7 Denis Diderot, UMR 1160 INSERM, Paris, France; ^2^ Université de Lorraine, CHRU-Nancy, Laboratoire d’Immunologie, Nancy, France; ^3^ Université de Lorraine, CHRU-Nancy, Département d’Anesthésie Réanimation Brabois Adulte, Nancy, France; ^4^ CHRU-Nancy, Plateforme de Cytométrie en Flux Diagnostique, Nancy, France; ^5^ Service de Médecine Intensive et Réanimation, CHU de Clermont-Ferrand, Clermont-Ferrand, France; ^6^ Université de Lorraine, INSERM UMR 1116, Nancy, France; ^7^ Université de Lorraine, CNRS UMR 7365, IMoPA, Nancy, France

**Keywords:** SARS-CoV-2, immunity, monocyte HLA-DR, antigen-specific polyfunctional T-cells, intensive care unit

## Abstract

Little is known about the time-dependent immune responses in severe COVID-19. Data of 15 consecutive patients were sequentially recorded from intensive care unit admission. Lymphocyte subsets and total monocyte and subsets counts were monitored as well as the expression of HLA-DR. For 5 patients, SARS-CoV-2–specific T-cell polyfunctionality was assessed against Spike and Nucleoprotein SARS-CoV-2 peptides. Non-specific inflammation markers were increased in all patients. Median monocyte HLA-DR expression was below the 8,000 AB/C threshold defining acquired immunodepression. A “V” trend curve for lymphopenia, monocyte numbers, and HLA-DR expression was observed with a nadir between days 11 and 14 after symptoms’ onset. Intermediate CD14^++^CD16^+^ monocytes increased early with a reduction in classic CD14^++^CD16^-^ monocytes. Polyfunctional SARS-Cov-2–specific CD4 T-cells were present and functional, whereas virus-specific CD8 T-cells were less frequent and not efficient. We report a temporal variation of both innate and adaptive immunity in severe COVID-19 patients, helpful in guiding therapeutic decisions (e.g. anti-inflammatory *vs.* immunostimulatory ones). We describe a defect in virus-specific CD8 T-cells, a potential biomarker of clinical severity. These combined data also provide helpful knowledge for vaccine design.

**Clinical Trial Registration:**

https://clinicaltrials.gov/, identifier NCT04386395

## Introduction

The SARS-CoV-2 outbreak causes a spectrum of clinical patterns that vary from asymptomatic but potentially contagious infection to mildly symptomatic and severe forms (1), suggesting a major role of the host response to SARS-Cov 2 virus. The severe form brings the patient to Intensive Care Units (ICU), with severe hypoxia frequently requiring mechanical ventilation (2). Until now, little is known about the relation between clinical patterns, systemic non-specific markers of inflammation and immune response. The previously reported modifications in severe forms of COVID-19 showed increased levels of C-reactive Protein [CRP], ferritin, lactate dehydrogenase [LDH]), associated with a marked lymphopenia of CD4 and CD8 T-cell subsets (3, 4). Innate immunity investigation reported normal absolute numbers of monocytes, with a reduced expression in their HLA-DR expression (5). Taken together, these modifications suggest an acquired immune-suppression, as reported in bacterial sepsis (6). Elevated levels of pro-inflammatory cytokines, mainly IL-6, have led to the hypothesis of an innate-mediated “cytokine storm” driving a systemic inflammation, neutrophilia and defective antigen-presentation (5). These dysregulations of both innate and adaptive immunity (7) explain apparent contradictions in COVID-19 therapeutic trials (8). We hypothesized that measurements performed only at the ICU admission do not represent the entire clinical picture, since the inflammatory process is varying along time for both innate and adaptive immunity. Consequently, we believe that a longitudinal monitoring may help to understand the complex immune reactions facing severe SARS-Cov2 infection, and be specially useful before decisions on immune-modulation therapeutics and establishing clinically relevant immune-monitoring (9).

This monocentric prospective study of severe ICU COVID patients reports a longitudinal evaluation of innate immunity based on monocyte subsets proportions and expression of HLA-DR (7, 10) and of adaptive immunity based on lymphocyte subsets absolute numbers (AN) and functions, referring to the onset of symptoms. Notably, we report on the presence of peripheral TH1-type SARS-CoV-2–specific T-cells in ICU COVID patients.

## Methods

### Patients

Among the enormous wave of severe cases admitted from March 30 to April 30, 2020 in east part of France, 15 cases of confirmed COVID-19 (positive RT-PCR for SARS-CoV-2 and suggestive chest CT-Scan) were prospectively investigated after ICU admission at the University Hospital of Nancy (CHRU-Nancy), France. The protocol was approved by the Innovation and Research Direction (reference 2020PI080), and by the Research Ethical Committee (Saisine 263) of CHRU-Nancy and registered at nih.gov (NCT04386395). No additional samples were drawn and no cells or plasma were stored after completion of the study. Relatives or patients themselves were questioned about objections to use the collected data for scientific purposes and/or potential publications. These statements and the non-opposition forms were dated and recorded in medical files.

After medical team consensus, patients did receive neither direct/indirect anti-viral treatment nor immunomodulating drugs except for 3 patients who received low dose steroids, allowing results interpretation based on a relatively pure COVID-19 natural evolution. Medical history, delay from the onset of symptoms and ICU admission (**Table S1**), classic clinical and routine biological data were recorded.

### Study Design

Complete blood cell evaluation was categorized according to time intervals from both the onset of symptoms and ICU admission for blood sampling as follows (**Figure S1**): A: days (d) 7–10 as a 1^st^ period (48 hours after ICU admission); B from d11 to 14; C from d15 to 18; D from d19 to 23; E: after d24. Similar analyses were performed referring to the delay from ICU admission: A: d0–4; B: d5–8; C: d9–12; D: late > d12 (**Table S2**). Also, in order to achieve a sufficient number of cases to test the relation between non-specific markers of inflammation with immune cellular response, the patients were alternatively grouped as follows: 1: days 7 to 14, 2: days 15 to 23, and 3: after day 24 (**Table 3**).

### Laboratory Investigations

Routine parameters, nonspecific inflammatory markers and immune-cells characterization were first measured on ICU admission (**Tables 1** and **2**) and these values were considered as baseline. Serial measurements were repeated until the patient was discharged or died. Flow-cytometry whole-blood routine analyses of circulating monocytes and lymphocytes were performed at the Diagnostic Flow-Cytometry platform of CHRU Nancy by using the BD FACSLyric™ Clinical System (BD Biosciences, San Jose, CA). All fluorochrome-conjugated antibodies and reagents were from BD Biosciences.

**Table 1:** Clinical characteristics, comorbidities, severity scores, nonspecific markers, metabolic parameters at the time of enrollment.

Variable	Value	Normal range
Number of patients	15	
*Patients’ characteristics*		
Age (y.o.)	66 [60; 72]/(54|75)	
Gender (male)	12 (80)	
BMI (kg/m²)	29 [24.5; 32]/(22|43)	
*Comorbidities*		
Diabetes	4 (26.7)	
Hypertension	7 (46.7)	
Obesity	6 (40)	
BMI	1 (6.7)	
COPD	2 (13.3)	
Asthma	1 (6.7)	
SAS	2 (13.3)	
Smoker	2 (13.3)	
Cancer	5 (33.3)	
On admission		
Delay 1st Symptoms	9 [7; 14]/(1|18)	
SAPS2	49 [42; 65]/(22|80)	
SOFA	7 [4; 8]/(4|12)	
SOFA resp	3 [3; 4]/(0|4)	
*Corticosteroids*	3 (20)	
*Nonspecific markers of inflammation*		
PCT (miss=2)	0.9 [0.5; 3.1]/(0.1|37)	
CRP (miss=1) mg/ml	174.4 [134; 284]/(85|389.2)	0;0–10.0
LDH	439.5 [397; 525]/(388|885)	120–246
Ferritin (miss=3) (µg/l)	888.5 [451; 2385]/(348|7200)	22–322
Ddimers (miss=1) (µg/l)	1742 [1599; 3220]/(804|10000)	45–500
Fibrinogen (miss=2) (g/l)	7.8 [6.5; 8.2]/(4.3|9.9)	1.7–4
C3 (miss=7) (g/l)	1.6 [1.4; 1.8]/(1.2|2.4)	0.9–1.7
C4 (miss=7) (g/l)	0.4 [0.3; 0.4]/(0.3|0.5)	0.12–0.36
*Metabolic parameters*		
Albuminemia (miss=1) (g/l)	25.5 [23.1; 27.4]/(18.2|31.5)	35–52
Cholesterol (miss=8) (g/l)	3.2 [2.5; 4]/(1.8|4)	< 2
TG (miss=4) (g/l)	2.1 [1.3; 2.4]/(1.1|2.8)	<1.5
Glycemia (g/l)	1.5 [1.2; 1.8]/(0.9|3.3)	<1.2
Lactate (mM/l)	1.3 [1; 1.5]/(0.8|1.6)	<2
*Outcomes*		
VAP	4 (26.7)	
Delay before VAP (miss=11)	13.5 [7; 15.5]/(1|17)	
ICU LOS	12 [10; 23]/(5|30)	
Death	3 (20)	

Data are expressed as medians [Interquartile IQ] or percentages (%). SAPS2: simplified acute physiological score; SOFA, sequential organ failure assessment; VAP, ventilatory acquired pneumonia; COPD, chronic obstructive pulmonary disease; SAS, sleep apnea syndrome; PCT, procalcitonin; LDH, Lactate dehydrogenase; CRP, C-reactive protein; C3, C4, complement fractions; TG, triglycerides.

**Table 2:** Time intervals of absolute and relative values referring to the onset of symptoms for blood lymphocytes, monocytes, polymorphonuclear cells and human leucocyte antigens-DR (HLA-DR, expressed in AB/C number of events per cell).

Variable	A : days 7–10	B : days 11–14	C : days 15-J18	D : days 19–23	E : days > 24	Normal range	Miss	P*All	P** A-B	P** A-D	P** A-E	P** B-D	P** B-E
Number of patients	5	9	12	11	9								
Leukocytes (109/l)	8.12 [7.83; 11.61]	10.79 [9.68; 11.23]	10.3 [6.8; 13.79]	11.48 [10.6; 15.19]	13.5 [11.7; 14.7]	4.0–10.0	5	0.09			0.04		0.04
PMNs (109/l)	6.26 [5.88; 7.09]	8.59 [7.79; 9.34]	6.12 [4.64; 9.07]	9.06 [8.03; 11.26]	9.5 [7.6; 11]	1.5–7.0	11	0.08		0.04	0.06		
Monocytes (109/l)	0.55 [0.34; 0.96]	0.52 [0.35; 0.6]	0.67 [0.27; 0.84]	0.61 [0.52; 0.79]	0.9 [0.7; 1]	0.2–1.0	3						0.04
Monocytes CD16- (%)	28.41 [21.26; 54.56]	69.72 [42.19; 83.76]	59.51 [41.16; 75.64]	53.26 [19.73; 66.88]	56.6 [44.1; 68.3]	80–85	7						
Monocytes Int (%)	67.51 [39.18; 72.95]	27.95 [14.57; 48.09]	37.81 [19.77; 53.92]	43.18 [30.11; 67.24]	35.9 [29.3; 53]	2–11	7						
Monocytes CD16+ (%)	3.04 [2.49; 5.3]	1.49 [1.06; 7.5]	2.52 [1.96; 3.77]	3.96 [1.62; 10.23]	3.7 [1.2; 7.7]	2–8	7						
HLA (AB/C)	6396 [3360; 6479]	3237 [2853; 4173]	4850 [4033.5; 7193]	5094 [4170; 7895]	8406 [3466.5; 11776]	16884[5842–29175]	1					0.08	0.08
HLA CD16- (AB/C)	4199 [2467; 6680]	2769 [1897; 4400]	3422.5 [2762.5; 5499.5]	4009.5 [2278.5; 6088]	4922.5 [2515; 6899]		7						
HLA Int (AB/C)	8805 [3684; 9510]	4758.5 [3457.5; 9677]	7907.5 [4984.5; 12331.5]	7883 [4416; 12552]	9623.5 [5756; 20052]		7						
HLA CD16+ (AB/C)	34565 [16673; 48542]	11864 [5352; 30733]	24179.5 [12204; 33577]	29197.5 [24749; 42757.5]	39806.5 [16598; 71223]		7					0.09	0.08
Lymphocytes	1.28 [1.18; 2.05]	0.54 [0.45; 0.91]	0.86 [0.63; 1.71]	1.07 [0.96; 1.44]	1.8 [1; 2]	1.0–3.0	2	0.03	0.07			0.03	0.02
LT4 (109/l)	0.64 [0.57; 0.66]	0.26 [0.2; 0.44]	0.46 [0.34; 0.68]	0.57 [0.45; 0.65]	0.8 [0.4; 1.1]	0.55–1.5	2	0.06	0.07			0.03	0.06
LT4 (%)	50 [27.8; 56.3]	54.45 [42.65; 57.25]	48.7 [41.6; 62.05]	47.9 [42.5; 53]	48.3 [45.3; 51.8]		2						
LT8 (109/l)	0.15 [0.11; 0.94]	0.07 [0.03; 0.1]	0.12 [0.04; 0.28]	0.19 [0.09; 0.2]	0.3 [0.1; 0.4]	0.3–1.3	2	0.02	0.08			0.02	<.01
LT8 (%)	11.8 [9.7; 46]	9.8 [6.1; 14.45]	14.85 [8.4; 16.1]	13.6 [8.8; 20]	16.2 [14.4; 19.2]		2						0.03
LB (109/l)	0.32 [0.21; 0.33]	0.14 [0.1; 0.21]	0.15 [0.06; 0.56]	0.21 [0.14; 0.25]	0.4 [0.2; 0.5]	0.09–0.6	2						0.05
LB (%)	17.8 [16.3; 20.4]	22.5 [19.85; 25.95]	20.1 [8.5; 33.1]	14.6 [11.1; 28.8]	20.9 [16.3; 26.3]		2						
L NK (109/l)	0.11 [0.11; 0.15]	0.09 [0.04; 0.14]	0.1 [0.05; 0.14]	0.15 [0.09; 0.23]	0.1 [0.1; 0.2]	0.15–1.1	2						
L NK (%)	8 [5.3; 8.2]	11.1 [8.35; 18.6]	11.8 [6.25; 14.5]	14.5 [7.6; 17]	7.7 [4.5; 10]		2			0.09			0.10
CD4/CD8	4.05 [0.62; 5.6]	5.26 [2.89; 8.57]	3.83 [2.5; 5.32]	2.78 [2.52; 5.02]	2.8 [2.4; 3.3]		2						0.10
Treg (109/l)	6.1 [5; 8.3]	7.85 [6.9; 10.15]	7.5 [6.5; 10.55]	9.15 [6; 14.9]	8.3 [6.5; 9.7]		3						0.88
PMNs/Lymphocytes	5.13 [2.72; 5.35]	15.88 [6.37; 19.06]	5.46 [2.91; 10.74]	6.85 [5.72; 11.13]	7.3 [3.4; 9]		13	0.05	0.02	0.06			016

Data are expressed as medians and interquartile [IQ]; LT4: CD4 T-cells; LT8: CD8 T-cells; LB: B-cells; L NK: NK-cells; Treg: Regulatory T-cells; PMNs: polymorphonuclear cells. The right part shows the variations observed over time for all cells (All) or between the periods of blood sampling: A, B, C, D, E. The comparisons between periods B and C, C and D, C and E did not show any significance. *p-value: Kruskall-Wallis test; **p-value: Wilcoxon test.

#### Innate Immunity

Whole-blood monocytes were selected as previously reported (**Figure S2**) (11). CD14-positive (+) cells with intermediate SSC were analyzed. Because of their specialized functions and phenotypes (10, 12), monocyte subsets were further analyzed based on CD14 and CD16 expression as classical (CD14^++^CD16^-^), non-classical (CD14^low^CD16^++^), and intermediate (CD14^++^CD16^+^) (13). Relative and absolute populations sizes were determined and HLA-DR expression was quantified as number per cell (antibodies bound per cell [AB/C] arbitrary units) using a commercial kit (Quantibrite™, BD Biosciences). Total monocytes HLA-DR median expression in healthy donors, using similar set-up conditions, was 16,884 (5,842–29,175) AB/C. Our laboratory’s threshold for acquired immunodepression diagnosis is 8,000 AB/C (11, 14). “Normal proportions” for monocyte subsets are 80% to 95% for classical monocytes, 2% to 8% for non-classical monocytes and 2% to 11% for intermediate monocytes (12, 13).

#### Adaptive Immunity

Routine whole-blood immunophenotyping of T, B and NK lymphocytes by flow-cytometry determined relative and AN of lymphocytes and their subsets, i.e. CD3^+^ T-cells, CD3^+^CD4^+^ T-cells, CD3^+^CD8^+^ T-cells, CD19^+^ B-cells, CD3^-^CD16^+^56^+^ NK-cells. Regulatory T-cells (Treg) were identified in whole blood as CD3^+^CD4^+^CD25^++^CD127^neg/low^ cells (15, 16). SARS-CoV-2–specific T-cell polyfunctionality (17, 18) against viral peptides was assessed in 5 patients to further explore COVID-19 adaptive immunity (patients #7, #9, #11, #12, and #13). Patients’ PBMC from 19 to 29 days after the onset of symptoms were stimulated *ex-vivo* by overlapping peptides covering protein sequences of the Spike and Nucleoprotein SARS-CoV-2 antigens (JPT Peptide Technologies, Berlin, Germany). Antigen-specific T-cells reactivity was assessed by intracellular IFN-κ, TNF-α and IL-2 production (19). Navios^®^ flow-cytometer and Kaluza^®^ software v2.1 (Beckman Coulter, Miami, FL) were used for data acquisition and analysis. As positive controls, PBMC were also stimulated by a mix of immunodominant microbial peptides (CEFX peptide pool; JPT Peptide Technologies) for diverse microbial antigens-specific TH1 responses. Antigen-specific CD4 and CD8 T-cells polyfunctionality was expressed by using the “Polyfunctionality Index” (PI) (18, 20). More details in reagents, flow-cytometry gating strategy used for the selection and quantification of Sars-Cov-2–specific T-cells are shown in **Table S3** and **Figure S3**.

### Statistical Analyses

Data were described as number (%) and median (interquartile range (IQR)) for categorical and continuous variables, respectively. Linearity of the variables were assessed *via* qqplot and Shapiro-Wilk tests. HLA was log10 transformed afterwards. To take into account the small samples sizes, comparisons relied on Fisher exact test for categorical data and Kruskal-Wallis or Wilcoxon tests for continuous data. Wilcoxon for paired tests were used to determine pair-wise differences. For the repeated measurements, summary statistics such as differences and median were initially used. Then, to assess and compare the variability of the variables over time, mixed-effects models were used. A p-value of less than 0.05 was considered significant. All analyses were performed using SAS software, version 9.4 (SAS) and R software, version 3.6.3.

## Results

### Cohort of Consecutive Patients

Forty six measurements in 15 severe COVID-19 ICU patients were performed. The median age was 66 years old [60-72], 80% male, with median BMI of 29 [24.5; 32] and hypertension in 46.7% (**Table 1**). The severity score were 49 [42; 65] for SAPS 2, 7 [4; 8] for SOFA (neuro component excluded), and SOFA_resp_ at 3 [3; 4] (**Table 1**). Comorbidities were present in 47% of the patients with pre-existent cancer (33.3%) and diabetes (26.7%) (**Table 1**). Because of hypoxia deterioration, all except 1 (**Table S1**) needed intubation and mechanical ventilation with a protective lung ventilation protocol. Importantly, none of the patients received any direct or indirect antiviral drugs nor specific immunomodulating drugs. Only 3 patients received low dose of prednisolone during their ICU stay (**Table 1**). Three patients died (20%) during the 2^nd^ or 3^rd^ week after ICU admission. All other patients were ICU discharged. A ventilator-acquired pneumonia according to classical CDC definition (21) was diagnosed in 26.7% of the patients.

### Laboratory Findings

**Figure 1:**
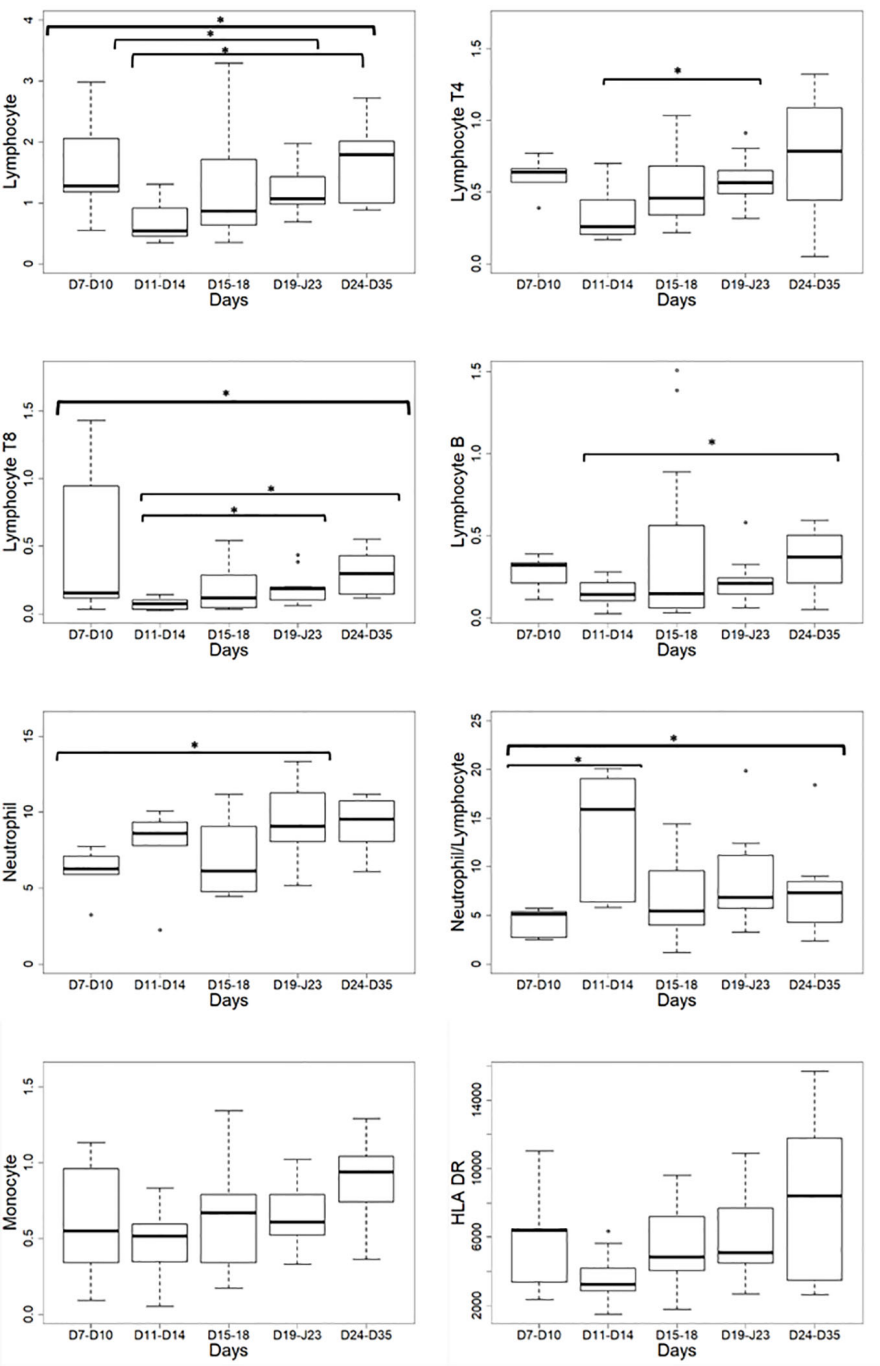
Leukocyte absolute number variations according to the delay from the onset of symptoms. HLA-DR: Human Leucocyte Antigen-DR expressed as events per cell (AB/C); *p < 0.05.

**Figure 2:**
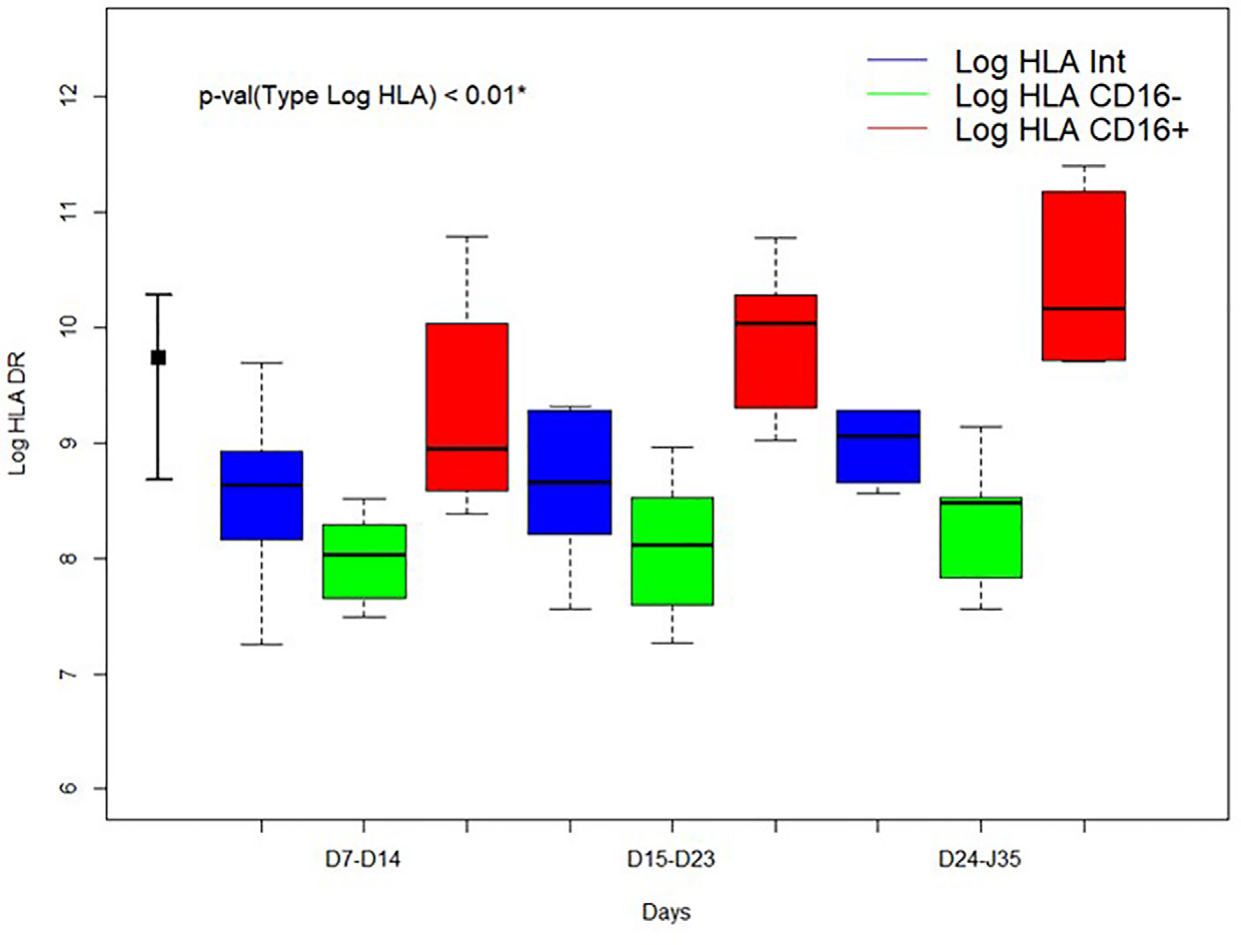
Time evolution of HLA-DR expression according to the delay from the onset of symptoms for 3 monocyte subtypes. Blue: intermediate (CD14^++^CD16^+^) monocytes; green: classical (CD14^++^CD16^-^) monocytes; red: non-classical (CD14^+^CD16^++^) monocytes. HLA-DR: Human Leucocyte Antigen-DR expressed as events per cell (AB/C). *Kruskall-Wallis test comparing the median values of HLA-DR expression of monocytes subtypes during all stay. Log: Log_10_. At left normal expected values for HLA-DR expression in total monocytes.

Non-specific markers of systemic inflammation such as CRP, ferritin, DDimers, fibrinogen, LDH, and the complement fraction C4 were largely above normal ranges in 100% of the patients at ICU admission (**Table 1**). DDimers levels were 3 times above the highest normal limit. Glycemia was slightly superior to normal values (1.5 [1.2; 1.8] g/L), with normal lactate levels (1.3 [1; 1.5]mM/L). Surprisingly, cholesterol (3.2 [2.5; 4] g/L) and triglycerides (2.1 [1.3; 2.4] g/L) levels were also largely above normal ranges, a lipid abnormality never reported in severe septic patients (22). Absolut numbers (AN) and relative values of leukocyte subsets are shown in **Table 2** and **Figure 1**. Considering the delay stratification from symptoms onset, there was globally no significant change in leukocyte AN (p= 0.09). Polymorphonuclears (PMN) initial AN were lower than the median observed at the later phases D (p = 0.04) and E (p = 0.06) (**Table 2** and **Figure 1**). A difference in monocyte AN was only significant when phase B (d11–14) was compared to the late phase E (p = 0.04) (**Table 2** and **Figure 1**). Importantly, the proportion of monocytes CD16+ intermediate was largely over the normal values (**Table 2**) (10, 23). Over time, the relative number for the 3 major subtypes of monocytes did not change significantly (**Table 3**). The ability of monocytes to express HLA-DR was lower than the threshold for acquired immunosuppression diagnosis at all periods, except for the latest measurement (>24 days after initial symptoms) (**Figure 2**). The lowest level of immunosuppression was observed at the initial phase d7–14 and tended to recover at the phase d15-D23 and d24–35, 14 days after symptoms, with an increase in proportion of CD16+ monocytes (p < 0.01) (**Figure 2**). Similar patterns were observed for all monocyte subtypes, albeit more pronounced for CD16^+^ monocytes (non-classical and intermediate) (**Table 3**). The association between modifications in monocyte proportions and HLA-DR expression and the nonspecific inflammatory markers is summarized in **Table 4**. No associations were found between the proportions of monocyte subtypes and the initial level (low or high value referring to the median) of CRP, DDimers, LDH and Ferritin (**Table 4**). For CRP, CD16+ intermediate monocytes increased the HLA-DR expression when the initial level was lower than the median value (p <0.02). Similarly when DDimers level was considered, both CD16+ and CD16+ intermediate monocytes increased their HLA-DR expression along time (p <0.04) when the DDimers level was lower than the median. Ferritin levels were also associated with the time dependent increase in HLA-DR expression in CD16- monocytes (p< 0.03) and CD16+ monocytes (p < 0.04). LDH levels were not associated with the changes in HLA-DR expression. No relation was found between the initial values of the nonspecific inflammatory markers and the lymphocytes subsets.

**Table 3:** Longitudinal cellular immune-inflammatory response referring to the delay from initial symptoms.

	A: Day 7 - 14	B: Day 15–23	C: Day 24–35	p-val All*	p-val A|B**
Number of patients	8	12	7		
Leukocytes (10^9^/l)	10.8 [9.7; 12.2]	11.7 [8.9; 14.7]	14.4 [12.9; 14.8]	0.08	–
PMNs (10^9^/l)	7.9 [7.7; 9.2]	9 [6.3; 11.3]	10 [8.5; 11]	–	–
Lymphocytes (10^9^/l)	0.6 [0.4; 1.2]	0.9 [0.6; 1.5]	1.4 [1; 1.9]	0.03	–
LB (10^9^/l)	0.1 [0.1; 0.3]	0.2 [0.1; 0.2]	0.3 [0.2; 0.4]	–	–
LT4 (10^9^/l)	0.3 [0.2; 0.6]	0.5 [0.3; 0.7]	0.6 [0.4; 1]	0.09	–
LT8 (10^9^/l)	0.1 [0; 0.1]	0.1 [0.1; 0.3]	0.2 [0.1; 0.3]	–	–
CD4/CD8	4.1 [1.6; 8.2]	2.8 [2.2; 5]	2.6 [2.4; 2.9]	–	–
L NK (10^9^/l)	0.1 [0; 0.2]	0.2 [0.1; 0.2]	0.1 [0.1; 0.2]	–	0.06
Treg (10^9^/l)	8.3 [5; 10]	7.1 [5.7; 9.5]	8.3 [6.2; 10.3]	–	0.06
PMN/Lymphocytes	10.8 [5.8; 19.1]	7.3 [5.2; 9.9]	7.6 [5.1; 9]	–	–
Monocytes (10^9^/l)	0.6 [0.4; 0.8]	0.7 [0.5; 0.9]	1 [0.7; 1.1]	0.09	–
Monocytes CD16- (%)	59.4 [42.4; 85.9]	64.3 [43.3; 73.2]	48.6 [44.1; 64.5]	–	–
Monocytes Int (%)	38.3 [14.6; 73]	53.9 [34.8; 62.2]	42.1 [29.8; 53]	–	–
Monocytes CD16+ (%)	1.6 [1; 9.9]	4.1 [2.6; 9.5]	5.9 [1.5; 7.7]	–	0.06
Log HLA (AB/C)	8.1 [7.9; 8.5]	8.5 [8.1; 8.7]	8.6 [8; 9.2]	–	–
Log HLA CD16- (AB/C)	8 [7.5; 8.3]	8.1 [7.6; 8.5]	8.5 [7.8; 8.5]	–	–
Log HLA Int (AB/C)	8.6 [8.1; 9.2]	8.7 [8.2; 9.3]	9 [8.7; 9.3]	–	–
Log HLA CD16+ (AB/C)	9 [8.5; 10.4]	10 [9.3; 10.3]	10.2 [9.7; 11.2]	0.1	–

Data are expressed as medians [Interquartile IQ] or percentages (%). PMNs: Polymorphonuclears; L : Lymphocytes; Log: Log_10_. *mixed-effects models; **Wilcoxon paired t test.

**Table 4:** Association of monocytes sub-populations and HLA-DR expression with nonspecific inflammatory parameters.

	Period	miss	Low CRP	High CRP	p*	Low Ddimers	High Ddimers	p*	Low LDH	High LDH	p*	Low ferritin	High Ferritin	p*
Monocytes CD 16 – (%)	A	2	57.7 [32.6; 84.8]	43.3 [42.2; 62.9]	–	56.1 [43.3; 84.8]	48.5 [37.4; 61.2]	–	0.8 [0.6; 1]	0.5 [0.3; 0.7]	–	0.7 [0.6; 1]	0.4 [0.3; 0.5]	–
	B	3	74.7 [36.9; 81.8]	21.5 [8.8; 42]	–	81.3 [31.6; 81.8]	36.9 [14.6; 68.1]	–	0.8 [0.6; 0.8]	0.6 [0.6; 1.1]	–	0.8 [0.6; 1.1]	0.6 [0.6; 0.7]	–
	B-A	3	4.4 [−3.5; 25.7]	−24.2 [−35.5; −0.4]	–	−3.2 [−3.5; 11.9]	−14 [−35.5; 7.2]	–	0 [−0.4; 0.2]	0.2 [0; 0.2]	–	0.2 [0; 0.2]	0 [0; 0.2]	–
	B-A>0	3	3 (50)	1 (16.7)	–	2 (40)	2 (28.6)	–	2 (50)	7 (77.8)	–	6 (75)	3 (60)	–
Monocytes CD 16 +(%)	A	2	1.8 [1.2; 4.4]	3.2 [1.7; 5.3]	–	2.3 [1.4; 4.7]	3.1 [1.4; 4.8]	–	84.8 [42.2; 85.9]	48.9 [32.6; 59.4]	–	54.6 [42.2; 62.9]	50.9 [31.8; 76.7]	–
	B	3	2.4 [1.6; 4.1]	9.7 [2.5; 10.6]	–	2.5 [2.2; 9.5]	4.1 [1; 10.6]	–	81.3 [6.7; 82.7]	36.9 [28.4; 68.1]	–	49.8 [11.7; 81.6]	39.4 [32.6; 61]	–
	B-A	3	−0.1 [−0.3; 0.2]	3.7 [0.3; 5.3]	0.09	0.2 [0; 2.2]	0.3 [−0.3; 5.3]	–	−3.5 [−35.5; −3.2]	−0.4 [−22.5; 11.9]	–	−3.3 [−35; 18.8]	−7.2 [−18.2; 3.4]	–
	B-A>0	3	2 (33.3)	5 (83.3)	0.08	3 (60)	4 (57.1)	–	0 (0)	4 (44.4)	–	3 (37.5)	1 (25)	–
Monocytes Int (%)	A	2	39.6 [14.6; 62.2]	49.6 [34.8; 54]	–	40.9 [14.6; 51.3]	44.4 [36.5; 58.1]	–	1.4 [0.3; 3.2]	3.7 [1.7; 5.3]	–	3.2 [1.7; 4.7]	2.1 [0.9; 5.1]	–
	B	3	21.1 [14.9; 57.1]	70.2 [46.6; 80.5]	0.07	15 [14.9; 57.1]	61.3 [27.3; 73]	–	2.5 [1.6; 11]	4.1 [2.2; 9.9]	–	6.8 [2.4; 10.6]	1.7 [0.9; 6.2]	–
	B-A	3	−3.1 [−25.9; 2.9]	20 [−3; 29.3]	–	0.4 [−6.5; 2.9]	12.6 [−7; 27.3]	–	2.2 [0.2; 7.8]	0 [−0.3; 2.7]	–	1.2 [−0.1; 5.1]	0 [−0.4; 1.5]	–
	B-A>0	3	3 (50)	4 (66.7)	–	3 (60)	4 (57.1)	–	3 (100)	4 (44.4)	0.09	5 (62.5)	2 (50)	–
Log HLA (AB/C)	A	0	8.6 [8.4; 9.2]	8.4 [8.1; 8.8]	–	8.5 [8.3; 9.2]	8.6 [8.1; 8.8]	–	14.6 [11.7; 54]	45.2 [38.3; 62.2]	–	40.9 [34.8; 54]	43.9 [21.6; 62]	–
	B	2	8.8 [8.5; 8.9]	8.3 [7.9; 8.5]	–	8.8 [8.5; 8.9]	8.2 [7.8; 8.6]	–	15 [14.6; 81.4]	57.1 [27.3; 67.5]	–	42.2 [14.9; 76.7]	54 [32.1; 64.4]	–
	B-A	2	0 [−0.4; 0.3]	−0.1 [−0.6; 0.1]	–	0.1 [0.1; 0.3]	−0.4 [−0.5; −0.1]	0.07	2.9 [0.4; 27.3]	−3 [−7; 23]	–	1.7 [−16.2; 28.3]	4.8 [−5; 17.8]	–
	B-A>0	2	3 (50)	3 (42.9)	–	4 (80)	2 (25)	0.05	3 (100)	4 (44.4)	0.09	5 (62.5)	2 (50)	–
Log HLA CD 16 – (AB/C)	A	2	8.4 [8.2; 8.5]	8 [7.8; 8.9]	–	8.2 [8.2; 8.5]	8.1 [7.8; 8.8]	–	8.5 [8.4; 8.6]	8.6 [8.1; 8.9]	–	8.7 [8.4; 9.2]	8.1 [8.1; 8.5]	0.10
	B	3	8.5 [8.3; 8.6]	7.9 [7.5; 8]	–	8.6 [8.3; 8.6]	7.8 [7.3; 8.5]	–	8.5 [8.5; 8.8]	8.4 [7.9; 8.9]	–	8.8 [8.5; 8.9]	7.8 [7.6; 8]	0.02
	B-A	3	0.1 [−0.2; 0.3]	−0.1 [−0.6; 0]	–	0.3 [0; 0.3]	−0.1 [−0.6; −0.1]	–	0 [−0.1; 0.4]	−0.3 [−0.5; 0.1]	–	0.1 [−0.1; 0.3]	−0.4 [−0.6; −0.3]	0.08
	B-A>0	3	3 (50)	1 (16.7)	–	3 (60)	1 (14.3)	0.10	2 (66.7)	4 (40)	–	6 (66.7)	0 (0)	0.03
Log HLA CD 16 + (AB/C)	A	2	9.7 [9; 10]	10.4 [10.2; 10.6]	–	9.7 [9.7; 10]	10.3 [9.7; 10.5]	–	8.2 [8; 8.5]	8.3 [7.8; 8.7]	–	8.3 [8.2; 8.5]	7.8 [7.7; 8.4]	–
	B	3	10.1 [10; 10.3]	10.3 [9.7; 10.6]	–	10.3 [10.2; 10.6]	10.1 [9.1; 10.5]	–	8.3 [7.8; 8.6]	8 [7.5; 8.6]	–	8.5 [8.2; 8.7]	7.4 [7.3; 7.8]	0.04
	B-A	3	0.4 [0.1; 0.6]	0 [−0.7; 0.1]	0.07	0.5 [0.3; 0.6]	0 [−0.7; 0.1]	0.04	−0.1 [−0.2; 0.4]	−0.1 [−0.5; 0.3]	–	0.1 [−0.1; 0.4]	−0.6 [−0.7; −0.3]	0.04
	B-A>0	3	5 (83.3)	3 (50)	–	5 (100)	3 (42.9)	0.04	1 (33.3)	3 (33.3)	–	4 (50)	0 (0)	0.08
Log HLA Int (AB/C)	A	2	8.9 [8.7; 9.3]	8.8 [8.2; 9.2]	–	8.8 [8.7; 9]	8.9 [8.3; 9.2]	–	9.7 [8.4; 10.2]	10.3 [9.7; 10.6]	–	10.2 [9.7; 10.6]	9.8 [9.1; 10.4]	
	B	3	9.5 [9.3; 9.9]	8.5 [8.1; 8.6]	–	9.5 [9.3; 9.9]	8.5 [7.8; 9.4]	0.10	10.2 [10.2; 10.3]	10.1 [9.7; 10.6]	–	10.2 [10.1; 10.6]	9.4 [8.8; 10.1]	0.10
	B-A	3	0.2 [0.2; 0.8]	−0.3 [−0.4; −0.1]	–	0.2 [0.2; 0.8]	−0.3 [−0.4; 0.2]	0.08	0.6 [0; 1.8]	0.1 [−0.6; 0.2]	–	0.2 [0; 0.6]	−0.3 [−0.7; 0.1]	
	B-A>0	3	5 (83.3)	1 (16.7)	0.02	4 (80)	2 (28.6)	0.08	2 (66.7)	6 (66.7)	–	6 (75)	2 (50)	

Data are expressed as medians [Interquartile IQ] or percentages (%). Miss: Missing values. Log: Log_10_.*p-value: Fisher exact test and Wilcoxon test as appropriate. Values B-A>0 refer for numbers of patients (% of total patients) for each monocyte sub-population. No significant results were observed within the lymphocyte subsets.

A significant positive correlation was found at all periods between HLA-DR expression and T-cells AN for CD4 (R^2^ = 0.28; p <0.01) and CD8 T-cells (R^2 =^ 0.22; p < 0.01) (not shown). Also, significant linear positive correlations were seen between HLA-DR expression on intermediate and non-classic monocyte subsets and CD4 T-cells AN (p <0.01) and between CD16^+^ intermediate subset and CD8 T-cells AN (p<0.01) (not shown). The analysis of the 3 patients who died did not suggest any specific patterns related to the poor prognosis, when compared to the surviving patients.

As reported (24), global lymphocyte counts showed a severe reduction in AN compared to normal ranges (**Table 2** and **Figure 1**). Lymphocyte AN changed significantly over time (p = 0.03). CD4 (“B” vs “D”: p = 0.03) and CD8 (“B” vs “D”: p = 0.02) T-cell AN were reduced at period B, being the “nadir” of lymphopenia, and then followed by a slow CD4 and CD8 T-cells increase (p<0.03) (**Figure 1**). The CD4/CD8 ratio was not impacted by lymphopenia. B-cells, NK-cells, and Treg numbers were stable along the monitoring time (**Table 2**). The leukocyte moderate elevation associated with the total lymphocytes rapid decline resulted in a significant rise in PMNs/Lymphocytes ratio at the “nadir” period B, with subsequent normalization (**Table 2**).

### Functional Analysis of Cellular Immune Response in SARS-CoV-2 Patients

**Figure 3:**
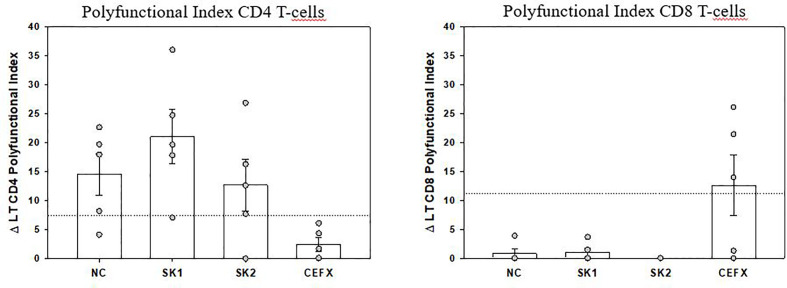
Polyfunctional Index (PI) of SARS-CoV-2–specific CD4 and CD8 T-cells. The PI of antigen-specific CD4 and CD8 T-cells from 5 patients was calculated as previously described (18, 20) for each antigen stimulation. Data are expressed after subtraction of “background polyfunctionality” of medium-stimulated cells (Δ PI). Dotted lines correspond to PI positivity threshold as determined at 3SD of 30 negative controls measures for CD4 and CD8 T-cells. Bars are shown as mean ± SEM. LT CD4: CD4 T-cells, LT CD8: CD8 T-cells.

**Figure 4:**
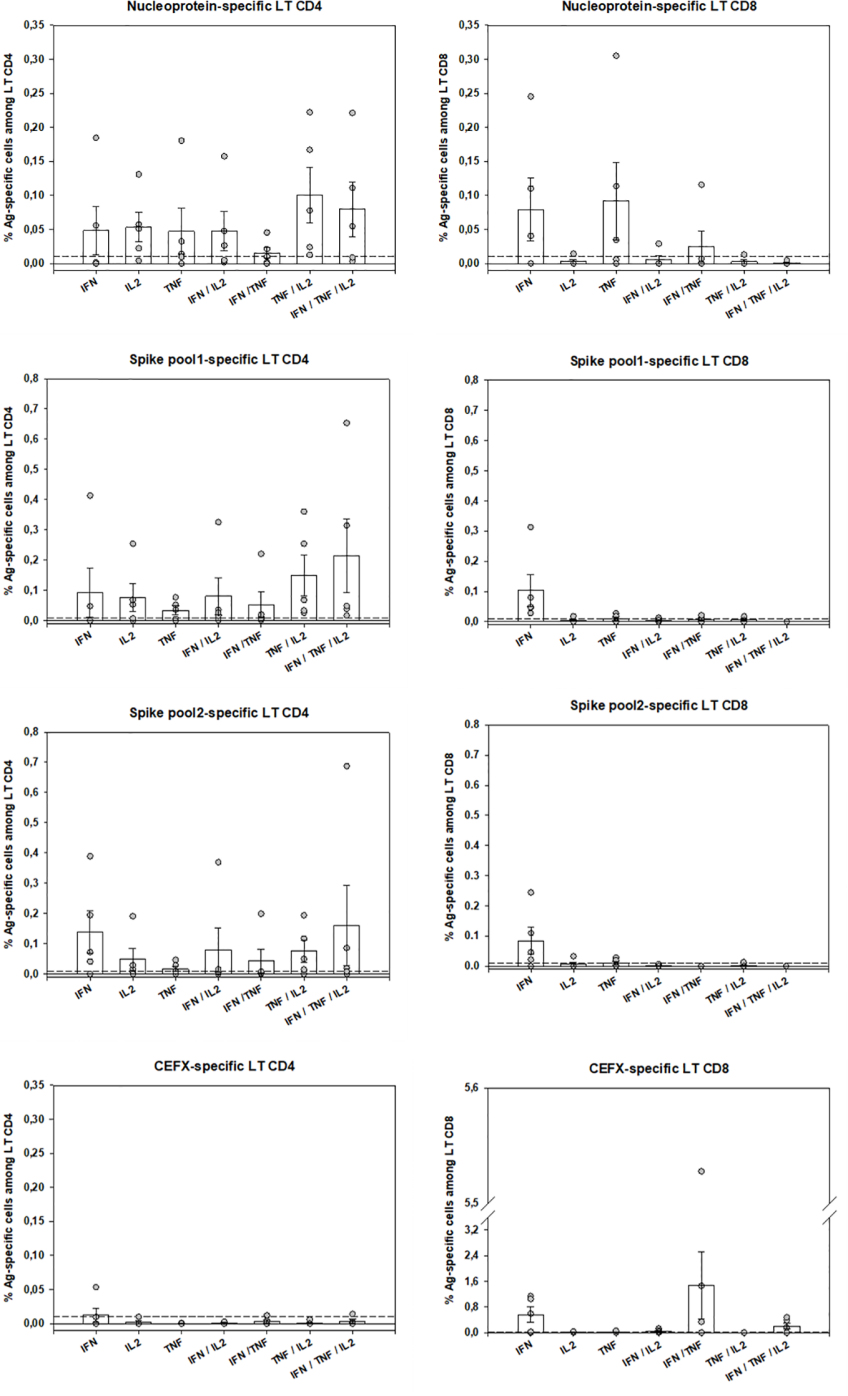
SARS-CoV-2–specific-T-cells numbers and functions. Peripheral antigen-specific CD4 and CD8 T-cells from 5 patients were identified by intracellular staining of IFN-κ, TNF-α and IL-2 by flow-cytometry as described (see Material and Methods and Suppl. Methods sections). Shown are individual data from stimulation with SARS-CoV-2 Nucleoprotein and Spike Glycoprotein peptides pools as well as by multiple immunodominant microbial peptides (CEFX pool) for CD4 and CD8 T-cells and for each cytokine capability (monofunctional, bifunctional or trifunctional). Basal cytokine production of medium-stimulated cells was subtracted for each antigen-stimulated cell subtype and a 0.01% positivity threshold was defined (dotted lines). Bars are shown as mean ± SEM. LT CD4: CD4 T-cells, LT CD8: CD8 T-cells.

Peripheral SARS-CoV-2–specific T-cells were identified in 5 patients by using an intracellular staining assay and flow-cytometry to evaluate the production of 3 TH1 cytokines contributive to viral clearance (17). PBMC were stimulated by 3 SARS-CoV-2 peptide pools (Sk1 and Sk2 pools and Nucleoprotein (NC pool)). The polyfunctionality Index (PI) for IFN-γ, TNF-α and IL-2 production showed that virus-specific CD4 T-cells were more polyfunctional than CD8 T-cells (**Figure 3**). Remarkably, a high proportion of bi-functional and tri-functional CD4 T-cells was observed, whereas CD8 T-cells were essentially monofunctional (**Figure 3**). Nucleoprotein-specific CD4 T-cells were less numerous than those specific for the Spike glycoprotein (both Sk1 and Sk2 pools), similarly to reported results (25). CD4 T-cells responses to immunodominant peptides from different infectious agents (CEFX pool) were much lower than CD8 T-cells responses (**Figure 4**).

## Discussion

The most significant predictors of disease severity in COVID-19 infection relate to both innate and adaptive immunity and their inter-relations. Until now, to our knowledge, no longitudinal clinical study on ICU-admitted patients has reported the evolution of monocyte subsets with concomitant HLA-DR expression and lymphocytes subsets AN. Precise knowledge of symptoms’ onset allowed us to interpret the collected data as related to disease progression, instead of admission time to ICU. Between symptoms’ onset and ICU admission to ICU discharge or death, we observed a “V” curve trend for monocytes and their HLA-DR expression as well as for lymphopenia, with a nadir between days 11 to 14. This 2 weeks timing already reported as a risk period for clinical events (1) was characterized by the lowest HLA-DR expression on monocyte subsets associated with the deepest CD4 and CD8 T-cells lymphopenia. The early intensity of inflammation characterized by the blood nonspecific markers levels seems to relate with the amplitude of immune modifications in monocyte subsets and their HLA-DR expression, concomitant with lymphopenia. The more these nonspecific inflammatory markers were initially elevated, the slower was the recovery of monocytes HLA-DR expression after the nadir 2 weeks post symptoms, an effect not yet reported in COVID 19 nor in septic patients. In this respect, the level of ferritin appeared the most promising marker with the addition of DDimers. Concerning the adaptative antigen-specific immunity, we found in a subset of patients that mainly polyfunctional SARS-Cov-2–specific CD4 T-cells were present 3 weeks after the onset of symptoms, while peripheral SARS-Cov-2–specific CD8 T-cells were less numerous and not efficient. However, a normal CD8 T-cells response for other microbial antigens was present, excluding a global functional defect of peripheral CD8 T-cells in these patients.

This ICU cohort had a predominance of males with high Body Mass Index and frequent comorbidities, as previously reported (26). Acute respiratory syndrome with severe hypoxia imposed intubation and mechanical ventilation for 14/15 patients. However, our cohort differs from others by several aspects: no patient received any anti-viral therapy or specific immunomodulatory drugs before and during ICU stay, avoiding a potential incidence on the immune profile. At referral to ICU, all patients had elevated levels of non-specific markers of systemic inflammation such as ferritin, LDH, and CRP. The mortality rate (20%) was relatively low and none of the patients experienced shock nor multiorgan failure. The reasons for that are not clear, except the reduced interference between drugs and immune status.

In absence of a longitudinal assessment, one-point checking for immune status has big limitations. As shown here, both innate and adaptive immunity vary over time after SARS-CoV-2 infection. COVID-19 severity is related to an initial excessive inflammatory response, pro-inflammatory cytokine storm and global lymphopenia as well as pulmonary mononuclear cell infiltration (27). The reported monocyte and macrophage hyperactivation have a major role on this hyperinflammatory state, potentially depending on their interaction with virus-specific T-cells (5, 28, 29). The virus itself, directly *via* pathogen-associated molecular patterns and indirectly *via* damage-associated molecular patterns, may activate multiple immune pathways (7).

If monocytes can initiate and amplify adaptative immune responses, they also play a key role supporting tissue homeostasis by resolving these responses to avoid excessive tissue damage (10). Monocyte classical (CD14^++^CD16^-^), non-classical (CD14^+^CD16^++^) and intermediate (CD14^++^CD16^+^) subsets reflect different functions (10, 23). Only one study has reported monocyte subsets proportions in 3 ICU COVID-19 patients (30). In our study, monocyte AN did not change along with COVID evolution in ICU, except when comparing the last measurements (>24 days) with those of days 11 to 14 after symptoms onset. Previous reports also showed maintained monocyte numbers (28). Proportions of the 3 monocyte subsets did not change significantly during the evolution period we studied. However, the intermediate CD14^++^CD16^+^ subset was remarkably always above reported normal values (10, 23), which was associated with reduced proportions of classic CD14^++^CD16^-^ monocytes, as reported (30). Functionally, HLA-DR global expression was below the 8,000 AB/C threshold defining acquired immunosuppression (11, 14), with similar trends for the 3 subsets. Such changes in monocyte proportions and functionality appear related to disease severity and suggest a maturation towards macrophages (28). The severe reduction in HLA-DR expression observed was comparable with that observed in bacterial sepsis (6) or trauma (14). The reported SARS-CoV-2 virus-induced restriction in interferon genes expression may account for severe reduction in different IFN proteins, including IFN-κ (29). The close correlation between CD4 and CD8 T-cells numbers and monocyte HLA-DR expression levels supports such hypothesis. The severe reduction in monocyte HLA-DR expression may also result from other mechanisms such as monocyte dysfunction, particularly secondary to exposure to IL-6 (5, 31), which requires further investigation.

COVID-19 mortality has been shown to correlate to global T-cell function as indirectly measured by T-cell lymphopenia (32). However, the successful containment of viral infections depends on the generation of antigen-specific TH1-polarized CD4 and CD8 T-cells, with a huge increase of effector T-cells at acute phase, which declines after successful virus control, yet maintaining an increased pool of pathogen-specific memory T-cells (33, 34). More than the quantity itself, anti-viral “T-cells quality” is widely recognized as the most important for successful anti-viral responses. It relates to the cells’ capacity to perform simultaneous anti-viral functions (e.g. cytokine or chemokine production, cytotoxicity, proliferation) (17, 18). Indeed, polyfunctional and monofunctional T-cells differ at molecular levels (35). At the time of writing, only 4 publications have assessed SARS-CoV-2–specific T-cells. Ni et al. described them in COVID-19 recovered patients, using only IFN-γ ELISpot, possibly underestimating the magnitude and breadth of the response and not discriminating CD4 or CD8 virus-specific T-cells (36). The other 3 studies identified by flow-cytometry T-cells upregulating activation markers (25, 37, 38). Although this “global activation” approach can potentially detect all antigen-specific T-cells, polyfunctional CD4 T-cells were not investigated. Grifoni *et al.* described only polyfunctional CD8 T-cells, assessed from recovered patients (38). Therefore, to date no study has assessed both CD4 and CD8 T-cell polyfunctionality in the COVID-19 context, especially on ICU patients. We believe that such evaluation is important to better understand immune responses against this new virus. Of note, in previous SARS-CoV epidemics, specific polyfunctional CD4 and CD8 T-cells were present several years after infection (39). The large combinatorial datasets of multiparametric flow-cytometry analysis of polyfunctionality can be integrated in a one-dimensional numerical tool, called polyfunctional index (PI) that integrates degrees and variations of cellular polyfunctionality (18, 20). Its application here showed that polyfunctional CD4 T-cells were present for at least one of the SARS-CoV-2 tested antigens in all 5 tested patients. Conversely, CD8 polyfunctional T-cells were not identified for any SARS-CoV-2 antigen in any patient. SARS-CoV-specific CD4 T-cells were mainly bi- (mostly TNF-α+IL-2+) and tri-functional (plus IFN-γ). Conversely, when present, the rare SARS-CoV-2–specific CD8 -reactive T-cells were mainly monofunctional (producing either IFN-γ or TNF-α). Of note, the CD8 T-cells reactive to the control mix of microbial peptides mentioned above (CEFX pool) were perfectly polyfunctional, indicating again that only the pool of peripheral SARS-CoV-2–specific CD8 T-cells is affected (**Figures 3** and **4**). This difference in quantity and quality between CD4 and CD8 SARS-CoV-2–specific T-cells might be explained by a preferential homing of the virus-specific effector CD8 T-cells to tissues, and especially the lungs and/or by T-cell functional exhaustion (40, 41). The latter hypothesis, that would imply selective PD-1 expression, is not supported by the good specific responses of helper CD4 T-cells in the same samples. Further evaluations of SARS-CoV-2–specific T-cells in bronchoalveolar lavage and of the expression of T-cell exhaustion inhibitory markers (e.g. PD-1 and TIM-3) are then warranted. Virus-specific CD8 T-cells are key in eliminating virus-infected cells (33) and Grifoni *et al.* have reported their presence in recovered patients (38). Combined with our findings, one may therefore hypothesize that inefficient SARS-CoV-2–specific CD8 T-cell responses could promote viral persistence and then virus-induced inflammatory damage in severe COVID-19 patients. Overall, SARS-CoV-2–specific polyfunctional CD4 T-cells were present at relatively high numbers, by comparison to other antigen-specific T-cells routinely investigated in our laboratory (42), suggesting a strong immune response. Further studies are needed to establish the kinetics of circulating SARS-CoV-2–specific T-cells over time and their relationship with COVID-19 clinical presentation.

In conclusion, we report the first concomitant and longitudinal evaluation of innate and adaptive immunity in COVID-19 severe cases in relation with admission values of blood nonspecific markers of inflammation. We believe that such extensive immunomonitoring studies are needed in order to accumulate a better knowledge in SARS-CoV-2 innate and adaptive immune-responses relationships. This approach might be helpful in making therapeutic decisions (e.g. anti-inflammatory *vs.* immunostimulation interventions) depending on the stage of the disease. This also will be of utmost importance for vaccine efficacy evaluation in future clinical trials.

## Data Availability Statement

The raw data supporting the conclusions of this article will be made available by the authors, without undue reservation.

## Ethics Statement

The studies involving human participants were reviewed and approved by Innovation and Research Direction (reference 2020PI080), and by the Research Ethical Committee (Saisine 263) of CHRU-Nancy. The patients/participants provided their written informed consent to participate in this study.

## Author Contributions

DP, M-RL, and MCB contributed on conceptualization and study design. MC, HM, CD, LP, M-RL, and MCB performed experiments, provided samples, and generated data. DP, ClD, and M-RL analyzed data. DP and MCB wrote the original draft. DP, ClD, M-RL, and MCB reviewed and edited the manuscript. All authors contributed to the article and approved the submitted version.

## Conflict of Interest

The authors declare that the research was conducted in the absence of any commercial or financial relationships that could be construed as a potential conflict of interest.
