# Supplementary material for: A Longitudinal Study of Immune Cells in Severe COVID-19 Patients
Source: Front Immunol. 2020 Oct 23;11:580250. doi: 10.3389/fimmu.2020.580250 (PMC7597438; doi:10.3389/fimmu.2020.580250)
Supplement: Supplementary file 1 [file DataSheet_1.docx]

**Supplement.**

**Supplementary methods.**

**Flow-cytometry assessment of** **SARS-CoV-2 specific T-cells.**

Peripheral blood was obtained by venipuncture and collected in heparin-containing tubes (BD Biosciences). Mononuclear cells (PBMC) were separated by density gradient no later than 6 hours after venipuncture, washed and counted prior to cell culture. PBMC (5x10^5^ to 1.5x10^6^ cells) were resuspended in 200µL of serum-free AIM-V medium and were separately stimulated overnight (12-14 hours) in 96 wells plate with peptide pools of overlapping 15-mer peptides (11 amino acid overlaps) spanning the entire Spike (divided in two pools of peptides: “SK1 and SK2”) and Nucleoprotein (pool “NC”) SARS-CoV-2 proteins (1.25 µg/mL of each peptide). Brefeldin A (Sigma-Aldrich) was added to each well and the plates set at 37°C, 5% CO_2_ humidified atmosphere. Unstimulated PBMCs (medium +DMSO, vehicle of the peptide pools) were used as negative controls. For positive controls of detecting antigen-specific responses, PBMC were separately stimulated with a control pool of 176 known immunodominant peptide epitopes of diverse infectious agents (CEFX pool). All peptide pools were from JPT Peptides (Berlin, Germany). All samples were also co-stimulated with antibodies directed against CD28 and CD49d (1 µg/mL, BD Biosciences). As positive controls of global TH1-immunocompetence, PBMC were also stimulated in parallel with Phorbol-Myristate-Acetate (50 ng/mL, Sigma-Aldrich) and Ionomycin (500 ng/mL, Sigma-Aldrich). After stimulation, cells were collected and washed before an incubation with BD Horizon™ Fixable Viability Stain 510 (FVS 510) and a cell surface antibody mix of anti-CD3 BB515, anti-CD4 PE-Cy7 and anti-CD8 PE-CF594. Cells were then fixed, washed and permeabilized according to the manufacturer’s instructions by using IntraStain® kit (Dako). Next, the cells were incubated with an intracellular antibody mix of anti-IL2 PE, anti-TNF-α APC and anti-IFN-γ BV421. All conjugated antibodies were from BD Biosciences. After 2 washes, cells were resuspended in PBS and immediately proceeded to flow cytometry data acquisition. Data were acquired using a Navios® Flow Cytometer (Beckman Coulter). SARS-CoV-2 -specific T-cells were identified using Kaluza® software v2.1 (Beckman Coulter) as by a specific gating strategy (Suppl. Figure3). The same gating strategy was used for SARS-CoV-2 Ag-stimulated cells and the percentage of Ag-specific CD4 or CD8 T cells was determined after subtracting the percentage of cytokine-positive events in unstimulated controls. Responses above the level of difference of 0.01% cytokine-producing cells were considered as Ag-specific positives (PMID: 19266489). In order to quantify the polyfunctionality of SARS-CoV-2 specific T-cells, we calculated the Special Polyfunctionality Index (PI) for each stimulation condition and for CD4 and CD8 T cells as described (1). We have used a higher ponderation for increasing polyfonctionnality (q=1.2) as determined in a series of statistical analysis correlating T-cell polyfunctionality and efficacy (2). Negative controls stimulation (medium plus co-stimulatory antibodies) present a background production of cytokines (mostly monofunctional cells), we then subtracted the Polyfunctional Index of negative controls from each specific antigenic stimulation in order to correct for this “background polyfunctionality” for each patient tested. The limit of detection for an augmentation of polyfunctionality was determined at 3 SD of results from 30 measures of negative controls for CD4 and CD8 of different subjects.

**Figure S1. Study design of sequential monocytes and lymphocytes characterization using flow-cytometry techniques.**


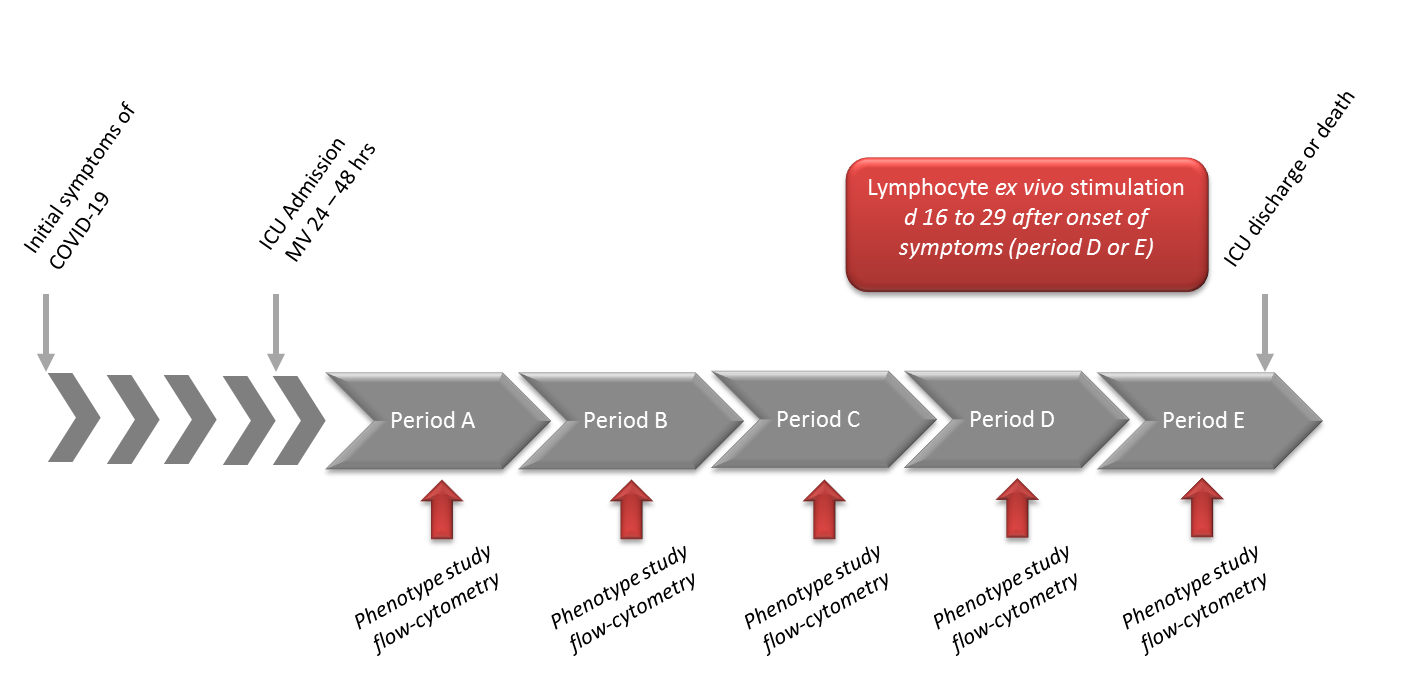


Suppl Figure S1 legend: Period A (d7-10), Period B (d11-14), Period C (d15-18), D (d19-23), Period E: > d24 after onset of disease. Ex vivo stimulation of lymphocytes (PBMC) with SARS-CoV-2 antigens was performed between d16 to d29. hrs: hours.

**Figure S2. Flow-cytometry sequential gating strategy for identification of circulating monocytes subsets.**


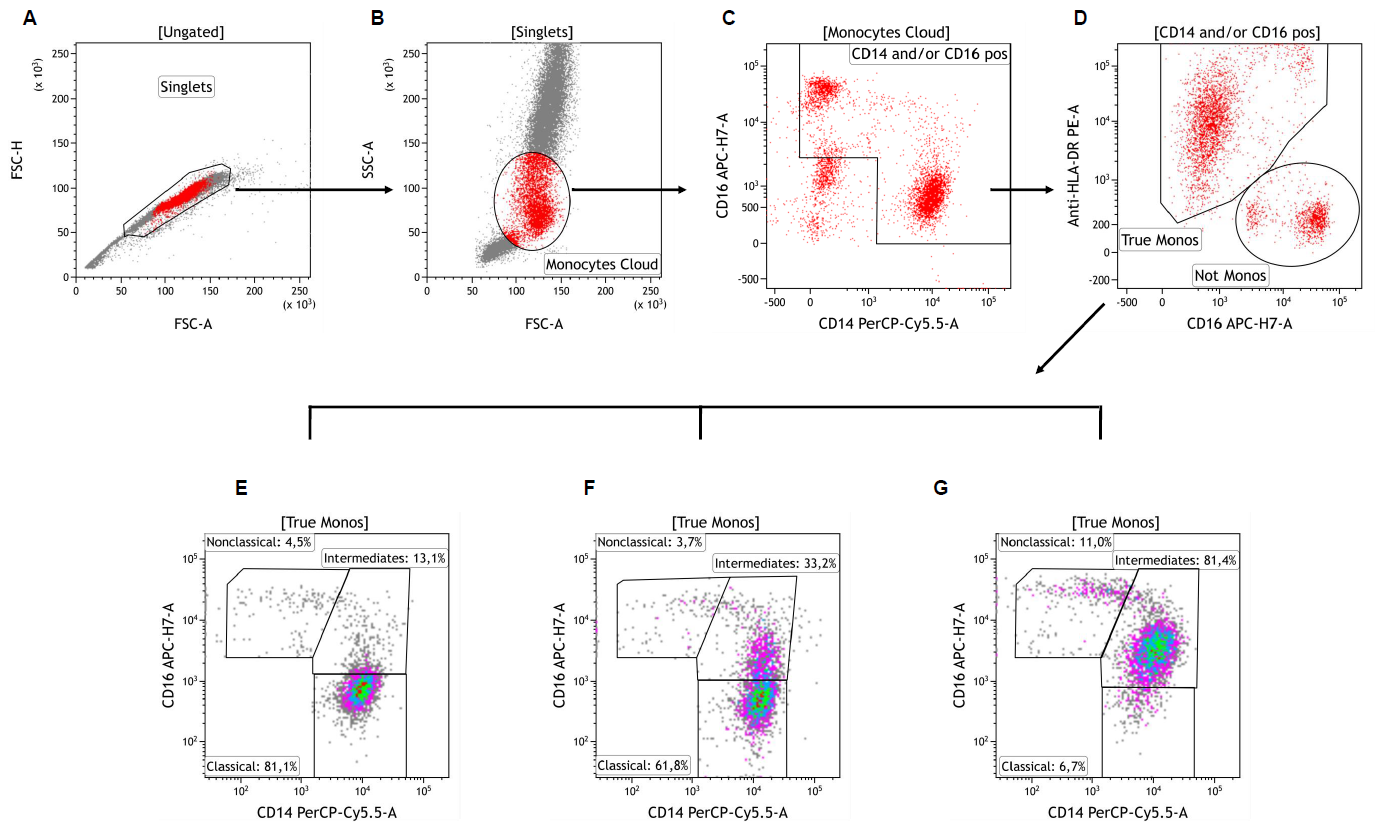


Suppl Figure S2 legend: Singlets were first identified on a FSC-A vs. FSC-H dot plot (Plot A). Cells were next visualized on FSC vs. SSC plot and an ample “Monocytes Cloud” gate was set, excluding most neutrophils and lymphocytes (Plot B). These cells were then viewed on a CD14 vs. CD16 plot and a gate was drawn excluding CD14 negative and CD16 low/negative cells (gate “CD14 and/or CD16 pos”, Plot C). Selected cells were next viewed on a CD16 vs. HLA-DR plot and the contaminating cells (‘‘Not Monos’’ gate) were easily distinguished from the ‘‘true’’ monocytes (“True Monos” gate, Plot D). These latter ‘‘cleaned’’ monocyte population was next viewed again on a CD14 vs. CD16 plot and gated as CD14++CD16- “Classical”, CD14++CD16+ “Intermediates” and CD14+CD16++ “Nonclassical” monocytes subsets. Plots E, F and G show representative examples of patients with low (Plot E), moderate (Plot F) and high (Plot G) proportions of Intermediate monocytes. Adapted from Abeles *et al.* (3).


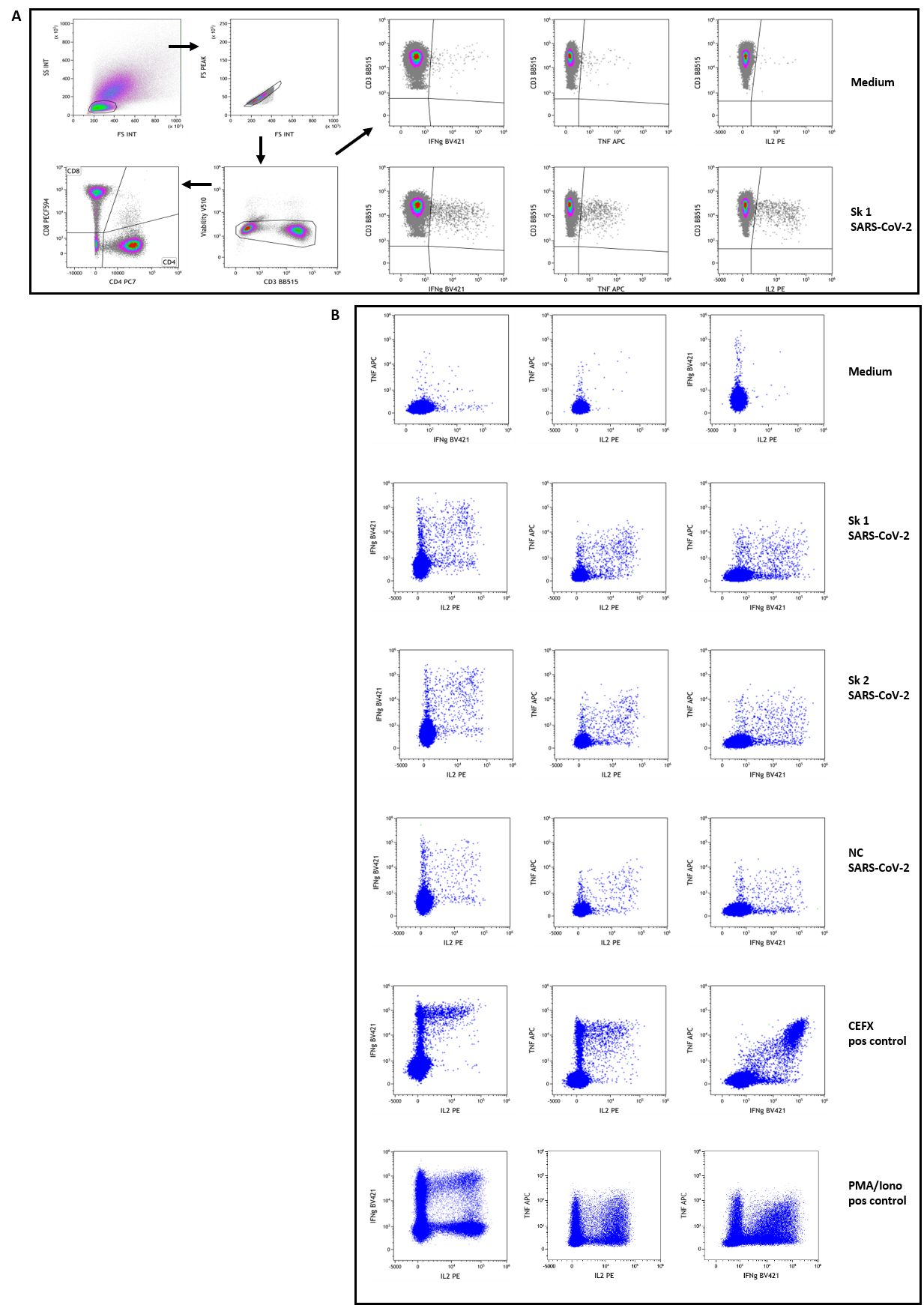
**Figure S3. Flow-cytometry assessment of SARS-CoV-2 specific T-cells.**

Suppl Figure S3 legend: Patients’ PBMC were stimulated overnight and then stained with fluorescent-conjugated antibodies to surface and intracellular molecules as described in Material and Methods section. Figure shows the gating strategy used to identify SARS-CoV-2 specific CD4 and CD8 T-cells in a representative subject (patient #13). Panel A: Live lymphocytes were defined after a sequential gating on FSC vs SSC, singlets and low FVS 510 staining. A gate was then set using the negative controls (medium stimulated cells) for the baseline expression of each cytokine as well as CD4 and CD8 subpopulations on total live CD3+ T lymphocytes. For cytokine-secretion assessment, boolean gates (not shown) were used to determine the frequencies of single-, double-, and triple-cytokine secreting CD4 and CD8 T-cells, defined as a percentage of IL2+, TNFα+ or IFNγ+ cells among selected T lymphocytes. These gates and analysis strategy were then set for all stimulation conditions for each patient assessed. Examples of stimulation with medium and peptides pool 1 of Spike SARSCoV-2 (Sk1) are shown at upper right. Panel B: Example dot-plots of bifunctional total T-cells identified are shown for medium (negative control), SARS-CoV-2 peptides pool 1 and 2 of Spike (Sk1 and Sk2) and Nucleoprotein (NC), as well as CEFX (multiple immunodominant microbial peptides pool) and PMA/Ionomycin positive controls stimulation. All shown data are from patient #13.

**Table S1. Clinical characteristics, context of COVID-19 disease, comorbidities and chronic treatment of the patients enrolled in the study.**

| *Patient N°* | *Age*  *(Yrs)* | *Gender* | *BMI* | *Medical history* | *Chronic treatment* | *Initial symptoms* | *Delay first symptoms -intubation (days)* | *Overall duration of MV (days)* | *LOS ICU*  *(days)* | *Pulmonary*  *embolism* | *Outcome*  *Alive = A*  *Dead = D* |
| --- | --- | --- | --- | --- | --- | --- | --- | --- | --- | --- | --- |
| 1 | 54 | M | 22 | no | no | Dyspnea  Cough | 8 | 8 | 10 | no | A |
| 2 | 62 | F | 31 | Hypertension, COPD, SAOS  Obesity | Sartan  Ca^2+^ channel blocker | Asthenia  Dyspnea | 7 | 12 | 18 | no | A |
| 3 | 60 | M | 24.5 | no | no | Dyspnea  Fever | 9 | 3 | 10 | no | A |
| 4 | 71 | M | 23.5 | no | no | Cough  Dyspnea  Fever  Diarrhea | 9 | 10 | 12 | no | A |
| 5 | 69 | F | 30 | Breast cancer  obesity | PPI  antidepressant  benzodiazepine | Fever | 8 | 27 | 30 | no | A |
| 6 | 69 | F | 25 | Hypertension  IDD, Stroke  cavernoma | Inuslin, Aspirine  Sartan, statins  antidepressant | Cough  Fever  Dyspnea | 5 | 9 | 11 | no | A |
| 7 | 73 | F | 33 | NIDD | metformin | Cough  Dyspnea  Fever  Diarrhea | 8 | 17 | 25 | no | A |
| 8 | 63 | M | 23 | Hypertension  Atheroma  COPD  Active smoking | Aspirin  PPI  Thiazide diuretics | Flu syndrome  Dyspnea | 16 | 18 | 21 | yes | A |
| 9 | 64 | M | 25 | Hypertension  ENT carcinoma | Calcium channel blocker  Alpha blocker | Dyspnea  Fever | 1 | 23 | 23 | yes | D |
| 10 | 72 | M | 30.5 | Hypertension  Asthma, COPD  Prostate cancer | Aspirin, Statins  Alpha and Beta blocker | Dyspnea | 14 | 14 | 14 | no | D |
| 11 | 65 | M | 27 | no | no | Cough  Fever  Dyspnea | 11 | 6 | 9 | no | A |
| 12 | 9 | M | 35 | Obesity  NIDD | Metformin; PPI | Dyspnea  Cough  Headache | 5 | 21 | 24 | yes | A |
| 13 | 65 | M | 43 | Obesity  IDD, CAD  Hypertension | Sartan -Ca^2+^ blocker - βblocker - diuretic -  Aspirin-Insulin  Statins | Asthenia  Dyspnea | 16 | 22 | 11 | no | D |
| 14 | 75 | M | 29 | Hypertension  High cholesterol  Smocker | Aspirin  β blocker  Hypolipemiant  CEI, lasilix | Dyspnea  Cough | 11 | 0 | 5 | no | A |
| 15 | 72 | M | 32 | Prostate cancer  Obesity  AA  SAS | Aspirin  Vitamin K antagonist  PPI | Dyspnea | 18 | 4 | 5 | no | A |

Suppl Table S1: Note the precise delay between the onset of symptoms and the ICU admission and intubation. ICU: intensive care unit; LOS: length of stay; MV: mechanical ventilation; SAS, sleep apnea syndrome; AA, aortic aneurysm; CAD coronary artery disease; COPD: chronic obstructive pulmonary disease; CEI: converting enzyme inhibitor; PPI, proton pump inhibitor.

**Table S2. Longitudinal immune parameters referring to the day of admission in Intensive Care Unit.**

| Variable | Miss | A: D 0 -4 | B: D 5-8 | C: D 9-12 | D: > D12 | Normal  range | p-val  All* | p-val  A-B** | p-val  A-C** | p-val  B-C** | p-val  C-D** |
| --- | --- | --- | --- | --- | --- | --- | --- | --- | --- | --- | --- |
| Number of patients |  | 9 | 12 | 12 | 6 |  |  |  |  |  |  |
| Leucocytes (10^9^/l) | 5 | 1.02 [0.53 ; 1.28] | 0.84 [0.55 ; 1.18] | 1.06 [0.91 ; 1.52] | 1.73 [1.41 ; 1.88] | 4.0-10.0 | 0.02 | - | - | - | - |
| PMNs (10^9^/l) | 10 | 0.14 [0.11 ; 0.28] | 0.16 [0.06 ; 0.23] | 0.2 [0.12 ; 0.25] | 0.37 [0.23 ; 0.58] | 1.5-7.0 | 0.06 | - | - | - | - |
| Monocytes (10^9^/l) | 2 | 0.41 [0.28 ; 0.96] | 0.61 [0.33 ; 0.79] | 0.59 [0.52 ; 0.83] | 0.88 [0.66 ; 1.04] | 0.2-1.0 | - | - | - | - | - |
| Monocytes CD16- (%) | 5 | 61.16 [48.36 ; 85.34] | 36.87 [28.41 ; 78.05] | 63.48 [42.83 ; 70.75] | 31.61 [30.61 ; 44.08] | 80-85 | - | 0.08 | - | - | - |
| Monocytes Int (%) | 5 | 36.52 [13.12 ; 46.61] | 61.32 [17.61 ; 67.51] | 32.98 [24.74 ; 50.42] | 53.93 [53.03 ; 57.07] | 2-11 | - | 0.08 | - | - | - |
| Monocytes CD16+ (%) | 5 | 1.54 [0.91 ; 3.11] | 2.55 [1.58 ; 5] | 3.96 [2.3 ; 7.48] | 9.46 [1.48 ; 10.62] | 2-8 | 0.08 | - | 0.08 | - | - |
| HLA (AB/C) | 0 | 5094 [4173 ; 6396] | 4481.5 [3211.5 ; 8595] | 4208.5 [2956 ; 7247] | 7887 [4782 ; 8933] | 16884[5842-29175] | - | - | - | - | 0.03 |
| HLA CD16- (AB/C) | 5 | 3997.5 [2661 ; 6214] | 3071 [2552 ; 5782] | 3063.5 [2006.5 ; 5131] | 5029 [4800 ; 6781] |  | - | - | - | - | - |
| HLA Int (AB/C) | 5 | 7762.5 [4915.5 ; 10029] | 5624 [4515 ; 11591] | 7136.5 [4576 ; 12552] | 10730 [10487 ; 10881] |  | - | - | - | - | - |
| HLA CD16+ (AB/C) | 5 | 21660 [9066 ; 31767] | 16673 [10992 ; 29499] | 24749 [16673.5 ; 31627] | 47671 [39954 ; 53627] |  | 0.03 | - | - | - | 0.06 |
| Lymphocytes (10^9^/l) | 1 | 1.02 [0.53 ; 1.28] | 0.84 [0.55 ; 1.18] | 1.06 [0.91 ; 1.52] | 1.73 [1.41 ; 1.88] | 1.0-3.0 | <0.01 | - | 0.05 | 0.05 | - |
| LT4 (10^9^/l) | 1 | 0.14 [0.11 ; 0.28] | 0.16 [0.06 ; 0.23] | 0.2 [0.12 ; 0.25] | 0.37 [0.23 ; 0.58] | 0.55-1.5 | 0.03 | - | - | - | - |
| LT4 (%) | 1 | 19.1 [10.4 ; 24.7] | 17.8 [8.7 ; 26.2] | 19.35 [12 ; 26.4] | 26 [14.6 ; 29.9] |  | 0.10 | - | 0.08 | - | - |
| LT8 (10^9^/l) | 1 | 0.07 [0.03 ; 0.11] | 0.11 [0.05 ; 0.15] | 0.15 [0.11 ; 0.19] | 0.09 [0.06 ; 0.23] | 0.3-1.3 | 0.02 | - | 0.08 | - | - |
| LT8 (%) | 1 | 6.5 [5.3 ; 10.4] | 12.6 [10.8 ; 15.4] | 12.95 [8.15 ; 20.6] | 6.45 [3.3 ; 14] |  | 0.03 | - | - | - | - |
| LB (10^9^/l) | 1 | 0.57 [0.31 ; 0.61] | 0.45 [0.28 ; 0.65] | 0.55 [0.35 ; 0.71] | 0.83 [0.5 ; 0.91] | 0.09-0.6 | 0.10 | - | - | - | - |
| LB (%) | 1 | 56.7 [50 ; 61.7] | 50.3 [45.9 ; 56.3] | 47.1 [41.45 ; 53.2] | 47.2 [40.8 ; 53.4] |  | - | - | - | - | - |
| L NK (10^9^/l) | 1 | 0.1 [0.03 ; 0.16] | 0.11 [0.04 ; 0.19] | 0.15 [0.1 ; 0.3] | 0.31 [0.18 ; 0.43] | 0.15-1.1 | 0.05 | 0.04 | 0.02 | 0.05 | - |
| L NK (%) | 1 | 11.8 [8.8 ; 15.8] | 13.1 [7.1 ; 14.9] | 16.3 [9.8 ; 17.7] | 16.6 [11.3 ; 22] |  | - | 0.01 | 0.02 | - | - |
| CD4/CD8 | 1 | 4.05 [3.76 ; 5.7] | 3.81 [2.74 ; 5.6] | 2.73 [2.23 ; 4.92] | 2.92 [1.83 ; 4.31] |  | 0.08 | - | - | - | - |
| Treg (10^9^/l) | 2 | 8.4 [6.1 ; 10.9] | 7.6 [6 ; 10.3] | 7.6 [7.1 ; 13.8] | 8.7 [6.5 ; 9] |  | - | - | 0.02 | - | 0.09 |
| PMNs/Lymphocytes | 12 | 19.1 [10.4 ; 24.7] | 17.8 [8.7 ; 26.2] | 19.35 [12 ; 26.4] | 26 [14.6 ; 29.9] |  | - | - | - | - | - |

Suppl Table S2: Values expressed in absolute and relative values of blood lymphocytes, monocytes, polymorphonuclear cells, human leucocyte antigens-DR (HLA-DR, expressed in AB/C number of events per cell) related to the time intervals referring to the ICU admission. Median and interquartile [IQ]; LT4: lymphocyte T4; LT8: lymphocyte T8; LB: B lymphocyte; L NK: lymphocyte Natural Killer; Treg: Regulatory T-cell; PMNs: polymorphonuclear cells. The right part shows the significance variations observed over time for all cells (All) or between the periods for blood sampling: A: D0-4, B: D5-8, C: D9-12, D: > D12. The comparison between B and C, C and D, C and E did not show any significance. Data are expressed as medians [Interquartile IQ] or percentages (%). Miss: Missing values. Log: Log_10_. * mixed-effects models ; ** Wilcoxon paired t test.

**Table S3. Fluorescent-conjugated antibodies and reagents used for flow-cytometry.**

| BD Quantibrite™ Anti–HLA-DR/Anti-Monocyte kit  (anti-HLA-DR PE, clone L243 + anti-CD14 PerCP-Cy5.5, clone MφP9) |
| --- |
| BD Quantibrite™ PE Fluorescence Quantitation Kit |
| BD Multitest™ 6-color TBNK Reagent with BD Trucount™ Tubes |
| CD16 APC-H7, clone 3G8 |
| CD3 - BB515 and APC, clone UCHT1 |
| IL-2 - PE, clone MQ1-17H12 |
| CD4 - PE-Cy7, clone SK3 |
| TNF-α - APC, clone MAb11 |
| IFN-γ - BV421, clone B27 |
| CD8 - PE-CF594, clone RPA-T8 |
| CD25- BV421, clone M-A251 |
| CD127 - PE, clone HIL-7R-M21 |
| Fixable Viability Stain 510 |

Suppl Table S3. All reagents from BD Biosciences, except CD3 – APC, from Biolegend.

**References.**

1. M. Larsen, D. Sauce, L. Arnaud, S. Fastenackels, V. Appay and G. Gorochov: Evaluating cellular polyfunctionality with a novel polyfunctionality index. *PLoS.One.*, 7(7), e42403 (2012)

2. A. Boyd, J. R. Almeida, P. A. Darrah, D. Sauce, R. A. Seder, V. Appay, G. Gorochov and M. Larsen: Pathogen-Specific T Cell Polyfunctionality Is a Correlate of T Cell Efficacy and Immune Protection. *PLoS One*, 10(6), e0128714 (2015) doi:10.1371/journal.pone.0128714

3. R. D. Abeles, M. J. McPhail, D. Sowter, C. G. Antoniades, N. Vergis, G. K. Vijay, E. Xystrakis, W. Khamri, D. L. Shawcross, Y. Ma, J. A. Wendon and D. Vergani: CD14, CD16 and HLA-DR reliably identifies human monocytes and their subsets in the context of pathologically reduced HLA-DR expression by CD14(hi) /CD16(neg) monocytes: Expansion of CD14(hi) /CD16(pos) and contraction of CD14(lo) /CD16(pos) monocytes in acute liver failure. *Cytometry A*, 81(10), 823-34 (2012) doi:10.1002/cyto.a.22104
